# Establishment and characterization of multicellular spheroids from a human glioma cell line; Implications for tumor therapy

**DOI:** 10.1186/1479-5876-4-12

**Published:** 2006-03-02

**Authors:** Divya Khaitan, Sudhir Chandna, MB Arya, BS Dwarakanath

**Affiliations:** 1 Institute of Nuclear Medicine and Allied Sciences, Brig. S. K. Mazumdar Marg, Delhi 110054, India; 2 SSV (PG) College Department of Zoology Chaudhary Charan Singh University Hapur, India

## Abstract

**Background:**

Multicellular spheroids, an appropriate *in vitro *system for simulating 3-D tumor micro-milieu can be used for evaluating and predicting tumor response to therapeutic agents including metabolic inhibitors. However, detailed understanding of the nature, distribution and sensitivity/responses of cellular sub-populations to potential therapeutic agents/strategies is required for using this unique model with optimal precision. Spheroid characteristics may also vary considerably with the origin and type of cell line used, and thorough characterization of viable and dissociated glioma cell spheroids is not yet completely known. In order to evaluate *in vivo *responses of gliomas to various therapeutic strategies, especially the metabolic inhibitors capable of penetrating the blood brain barrier, we have characterized continuously growing spheroids of a human glioma cell line (BMG-1) with respect to organization, growth, viability, cell survival, cell death, metabolic and mitochondrial status, oxidative stress and radiation response using microscopy, flow cytometry and enzymatic assays. Spheroids were fed daily with fresh medium in order to maintain nutrient supply to outer cellular layers while hypoxia/necrosis developed in the innermost cells of enlarging spheroids.

**Results:**

Volume of spheroids, fed daily with fresh medium, increased exponentially during 7–28 days of growth through three population doublings. Proportion of G_1_-phase cells was higher (~60%) than exponentially growing monolayer cells (~48%). A significant fraction of S-phase cells turned metabolically inactive (disengaged in DNA synthesis) with increasing age of the spheroids, unlike in quiescent monolayer cultures, where the fraction of S-phase cells was less than 5%. With increasing spheroid size, increasing sub-populations of cells became non-viable and entered apoptosis or necrosis revealed by Annexin-V-FITC/PI staining. PI positive (necrotic) cells were not confined to the centre of the spheroid, but distributed at certain discrete foci. Average glucose consumption and lactate production were 2–3 folds higher in viable spheroid cells compared to monolayer cells, implying a compensatory increase in glycolysis possibly due to hypoxic environment. HIF-1α was expressed only in spheroids and increased in an age-dependent manner, whereas c-Myc (known to induce apoptosis in glucose-deprived cells) levels were three times higher than monolayer cells. Mitochondrial mass and activity decreased significantly during first 14 days of growth but increased with age, and were not associated with increase in ROS levels. Bcl-2 and Bax levels were higher (~2 folds) than monolayers, while the ratio (Bcl/Bax) remained unaltered. Radiation-induced oxidative stress was considerably less in spheroids as compared to monolayers, and corresponded well with increase in radioresistance demonstrated by the clonogenic assay, similar to hypoxia induced radioresistance observed in tumors.

**Conclusion:**

Development of S-negative cells and reduced endogenous and radiation-induced ROS coupled with higher levels of anti (Bcl2) as well as pro (Bax) apoptotic regulators observed in spheroids suggest the intricate/complex nature of endogenous as well as induced stress resistance that could exist in tumors, which contribute to the treatment resistance.

## Background

Cells of actively growing tumors are exposed to varying micro-environmental conditions due to non-homogeneous vascular supply [1], leading to the development of localized regions in tumors having low oxygen tension, low glucose concentration and acidic extracellular pH due to accumulation of metabolic by-products such as lactic acid. As a result, cells in these regions are exposed to varying levels of hypoxia, anoxia and acidosis [2]. From the radiobiological point of view, hypoxic cells constitute the most important cellular subpopulation because they are more radioresistant than the euoxic cells as a result of reduced generation of radiation-induced reactive oxygen species or ROS [3,4]. The hypoxic cell fraction increases considerably with the advancement of tumor size and grade offering significant resistance to radiotherapy. The currently used hypoxic cell sensitizers (nitroimadazole compounds) have met with limited success due to the lack of differential effect besides systemic toxicity. Therefore, there is a need to develop effective strategies to selectively enhance the radiosensitivity of these cells, which requires a detailed characterization of the nature and responses of these resistant cellular fractions. Since hypoxic cells exist in three-dimensional tumors that behave as heterogeneous systems demonstrating alterations in many vital genotypic and phenotypic characteristics regulating various biological processes, an *in vitro *cellular model that closely simulates these conditions is essential for carrying out studies on hypoxic cell and tumor responses to therapeutic agents.

Monolayer cultures of established cell lines from human tumors have been widely used for studying the various molecular processes including the identification of specific molecular lesions related to the dysregulation of cell proliferation and cell death, the two important functional targets for the development of therapies. Unfortunately, investigations necessary for the development and/or evaluation of some of the therapeutic strategies cannot be carried out with this most widely used laboratory system, since complexities arising out of 3-dimensional organization of solid tumors (viz., cell-cell/cell-matrix interactions and variations in vasculature and nutrient supply) resulting in subtle changes in phenotypic expression (especially the metabolic changes) are not provided in the monolayer cultures derived from tumor cells. In contrast, multicellular spheroids of tumor cells provide an excellent three-dimensional *in vitro *model in which hypoxic conditions can be generated to facilitate detailed investigations including the response to various chemical agents and radiation [5,6].

Spheroids are characterized by high cell-density and a closely packed, 3-D tumor like structure, which leads to severe diffusion limitations for molecules as small as glucose and oxygen, thereby creating heterogeneous cell sub-populations of actively proliferating as well as quiescent, hypoxic and necrotic cells as found in solid tumors [7]. Available evidences suggest that low concentration of glucose and oxygen in the inner regions of spheroids may contribute to the formation of quiescent, hypoxic, anoxic and necrotic cell sub-populations [8-10]. Characterization of the nature of these cell sub-populations as well as their responses to radiation and radiomodifying agents can help in the development of more effective radiosensitizing strategies. For example, hypoxic cells are known to derive a large part of their energy from glucose-dependent anaerobic metabolic pathways. Therefore, inhibitors of glycolysis could specifically modify the responses of this resistant sub-population of cancer cells. A number of *in vitro *studies have indeed shown that 2-DG, a glucose analogue and inhibitor of glycolytic ATP production [11-13], selectively inhibits energy-dependent DNA repair and cellular recovery processes in cancer cells [14-16] resulting in enhanced cell death [11,13,17,18]. Spheroids could serve a very useful model to understand further the mechanisms of radiosensitivity induced by this glycolytic inhibitor in solid tumors, since spheroids mimic the solid tumors more closely than the monolayer culture.

Spheroids have been used for a broad spectrum of studies in cancer biology and employed extensively in radiobiological investigations [19] as they provide a good *in vitro *system to mimic the radioresistant hypoxic cell population generally found in tumors. Unfortunately however, detailed information available on the intercellular variation in mitochondrial mass, activity and ROS levels (oxidative stress mainly responsible for damage induced by low LET radiation) as a function of spheroid growth and with reference to other parameters like viability cell death etc in culture is lacking. These parameters influence the end results of *in vitro *studies aimed at understanding tumour response to various cytotoxic agents and metabolic inhibitors. Therefore, the present study was undertaken to systematically characterize spheroids established from a human glioma cell line BMG-1 [11,20] with respect to organization, growth, viability and cell survival, cell death, metabolic and mitochondrial status, oxidative stress and radiation response with age and spheroid size using microscopy, flow cytometry and enzymatic assays.

The results of the present studies demonstrate development of S-negative cells, reduced endogenous and radiation-induced ROS besides changes in levels of anti (Bcl2) as well as pro (Bax) apoptotic regulators observed in spheroids which suggest the intricate/complex nature of endogenous as well as induced stress resistance that could exist in tumors, which contribute to the treatment resistance.

## Results

### Growth

**Figure 1:**
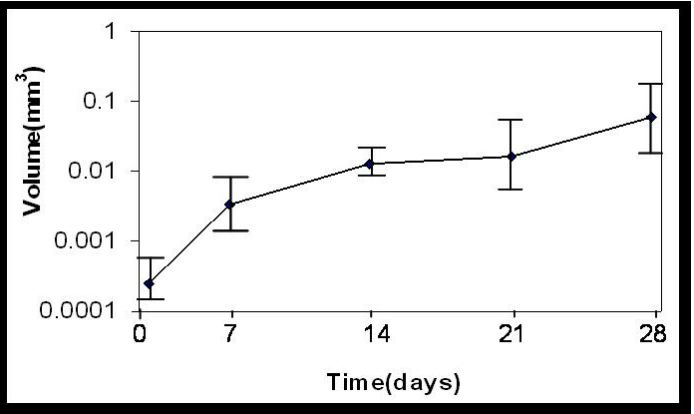
**Spheroid growth**. Growth of the BMG-1 spheroids revealed by the increase in the volume of spheroids as a function of time.

**Figure 2:**
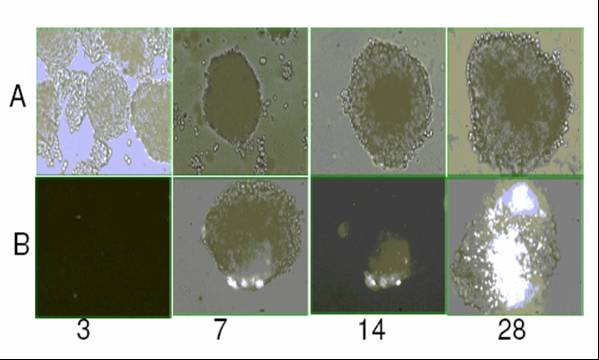
**Age dependent cell viability**. Photomicrographs of unfixed spheroids showing increase in size and dead cell population. Panel A: Bright field images of unstained spheroids. Panel B: Propidium iodide stained spheroids.

The growth of spheroids was monitored by measuring the volume and surface area of at least 50 spheroids at intervals of 7 days. The initial aggregation of monolayer cells plated for spheroid formation lasted for three days. Within 6–7 days the small clumps were replaced by compact spheroids with a radius of 80–100 micro meters. The volume vs. time plot of spheroids showed an exponential growth pattern of spheroids (Fig 1) till 28 days. Unlike spheroids from other cell lines, BMG-1 spheroids did not show any plateau phase in the growth curve as they disintegrated between 27–29 days, thereby necessitating a detailed characterization before initiating any further work. Despite this exponential increase in size, the phase contrast images acquired at different time intervals (3, 7, 14 and 28 days) showed an enhanced proportion of dead cells at the center of spheroids, which increased with age (Fig 2). The viability (assessed using trypan blue dye exclusion assay) decreased from 93% in 7-day-old spheroid to 70% in the 28-day-old spheroids. The clonogenic potential (plating efficiency) also decreased from 65% in the 7-day-old spheroids to 25% in the 28-day-old spheroids, indicating an increase in cell death with age (Table 1). This is in contrast to the plating efficiency of the monolayer BMG-1 cells, which was 85% as reported earlier [21].

**Table 1:** Spheroid size and cellular characteristics as a function of age in spheroids.

Age of spheroids in days	Radius (μm)	Av. volume × 10^-6^(μm^3^)	Avg. No. of cells/spheroid	Avg. Cellular Diameter (μm)	Cell packing density	Viability (%)	Plating Efficiency (%)
7	93 ± 5	3244	1176 ± 39	13 ± 1	0.36	98 ± 1.4	65 ± 0.6
14	148 ± 10	13300	2222 ± 47	7 ± 1(20%); 14 ± 1	0.17	78 ± 1.9	63 ± 0.2
21	157 ± 11	15896	4236 ± 63	10 ± 1 (25%); 16 ± 1 (63%); 24 ± 2 (12%)	0.27	77 ± 1.7	45 ± 0.2
28	242 ± 19	57891	8975 ± 78	19 ± 2	0.16	70 ± 3.2	25 ± 0.1

Note: All values are mean ± SD of 20–50 observations from each experiment (radius, surface area, volume, Number of cells/spheroid and average size of cells) or 6 observations (viability and plating efficiency) from three experiments.

### Cell cycle distribution

**Figure 3:**
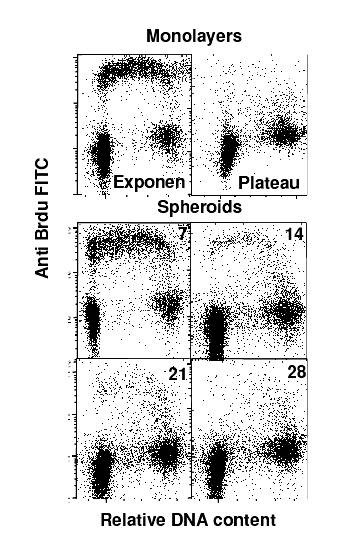
**Cell cycle distribution**. Cell cycle distribution of BMG-1 spheroids and monolayer cells.

To determine whether the cessation of cell growth in the spheroid culture was similar to the arrest of cells in a particular phase of the cell cycle as is generally observed in the stationary monolayer, we analyzed the fraction of active S-phase cells (BrdU label) and the DNA content by the flow cytometry. Fig 3 shows bivariate flow cytometric dot plots of DNA vs BrdU for BMG-1 monolayers and spheroidal cells showing cell cycle distribution of cells labelled with BrdU. Interestingly, a consistently higher proportion of cells were in G1 phase (60%) and significantly reduced S phase (20%) and G_2 _phase (20%) was observed throughout the growth of the spheroids (7–28 days) as compared to monolayers (G_1 _50%, S 40% and G_2_M 12%) in exponential phase. In spite of S phase being constant, the percent of BrdU positive cells (actively proliferating cells) decreased drastically from 18% in 7 day old spheroid to 5% in 28 day old spheroid. However there was an increase in S_0 _cell population (quiescent S phase cells) with the age of spheroid in culture, therefore, showing constant S phase cells throughout the growth of spheroid in culture (Table 2).

**Table 2:** Comparison of cell cycle distribution in exponentially growing monolayers and spheroids studied as a function of age.

Age of spheroids in days	G_0_/G_1_	Total S Phase Cells	S-Positive Cells	S-Negative Cells	G_2_/M
Monolayers	48 ± 3	40 ± 1	40 ± 1	0	12 ± 1
Plateau	72 ± 2	3 ± 2	3 ± 2	0	25 ± 4
07	59 ± 2	20 ± 3	18 ± 3	2 ± 0.5	21 ± 2
14	59 ± 2	20 ± 1	9 ± 1	11 ± 1	21 ± 1
21	56 ± 3	19 ± 2	6 ± 2	13 ± 1	25 ± 3
28	58 ± 4	19 ± 1	5 ± 1	14 ± 1	23 ± 1

Note: All values are mean ± SD of 3 observations from three experiments

### Cell death

**Figure 4:**
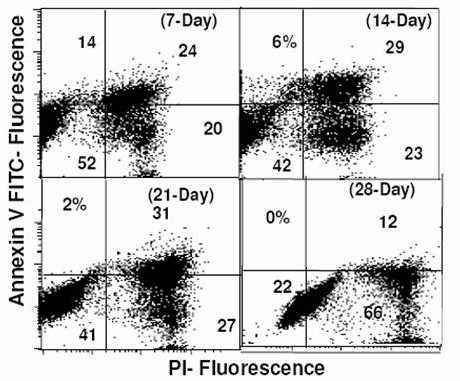
**Apoptotsis by flow cytometry**. Bivariate plots of Propidium iodide staining (revealing non-specific membrane damage) and Annexin V binding (PS externalization) studied by flow cytometry showing the dynamics of apoptotic cells as a function of age in culture.

Staining with annexin V has been used as a specific marker for cells in the early phase of apoptosis where the cell membrane is still intact. Asymmetry of plasma membrane that often occurs during apoptosis is mainly due to the translocation of phosphotidyl serine from inner leaflet of the membrane to the outer surface of the cell revealed by the binding of Annexin V [22]. On the other hand, non-specific membrane damage is associated with necrotic death indicated by permeability to PI. To characterize the nature of cell death, we analyzed the spheroids for the presence of apoptotic and necrotic, the two main forms of cell death. Dual staining with Annexin V FITC and PI clearly showed an increase in Annexin positive and PI positive cells indicating accumulation of apoptotic as well as necrotic cells with age upto 21 days (Fig 4). Interestingly, however the fraction of Annexin-V positive cells decreased significantly at 28 days, while a profound increase in the necrotic cell population (PI positive cells; >78%) was evident. These changes in the fraction of dead cells (apoptotic and/or necrotic) correlated reasonably well with the fraction of clonogenically viable cells (Table 1).

### Glucose usage and lactate production

Development of hypoxic regions with increase in spheroid cells is expected to result in enhanced glucose usage and anaerobic glycolysis, partly responsible for alterations in oxidative status, therefore, glucose usage and lactate production in BMG-1 spheroids and monolayers were studied by estimating the residual glucose and the lactate production at the end of 2-h incubation in HBSS. In the 7-day old spheroids, the glucose consumption (2.1 p mole/cell/h) as well as lactate production (3.67 p mole/cell/h) was nearly 2–3 fold higher than the monolayer cells (0.83 & 1.47 p mole/cell/h respectively, (Table 3), clearly suggesting a relatively more hypoxic environment in the spheroids. Growth of spheroids upto 28 days yielded further increase in lactate production, resulting in a significant reduction in pH of growth medium (data not shown), thereby resembling the intratumoral acidic condition.

**Table 3:** Comparision of glucose usage lactate production and radiation response in the exponentially growing monolayers and as a function of age in spheroids.

Model	Glucose utilization (pico mole/cell/h)	Lactate production (pico mole/cell/h)	D_q_(Gy)	D_0_(Gy)	S.F2
Monolayer	0.83 ± 0.03	1.47 ± 0.01	1.2	2.4	0.8 ± 0.04
7 day spheroid	2.10 ± 0.07	3.67 ± 0.04	1.8	3.2	0.9 ± 0.03
28 day spheroid	1.85 ± 0.05	2.93 ± 0.02	2.8	2.7	1.0 ± 0.06

Note: All values are mean ± SD of 6 observations from two experiments

### Alterations in HIF-1α, c-Myc, Bcl2 and Bax protein levels

**Figure 5:**
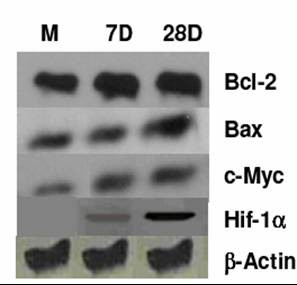
**Protein analysis**. Western blots analyses of different proteins Bcl2, Bax, c-Myc and HIF-1α in BMG-1 monolayers and spheroids.

Since the glucose utilization and lactate production was significantly higher in spheroids as compared to monolayers and hypoxia could be one of the contributing factors for enhanced glycolysis, we examined the levels of HIF-1α in these two model systems using immunoblotting. As expected, the HIF-1α could not be found in the monolayer cultures, while age-dependent increase in its level was evident in the spheroidal cells (Fig. 5), suggesting thereby that the enhanced glycolysis could be partly due to HIF-1α induced over expression of certain glycolysis-related genes on account of hypoxia. These observations are in agreement with the earlier findings showing the presence of hypoxic cells in spheroids [5]. Stress-induced apoptosis (particularly glucose deprivation) in cells with up-regulated glycolysis has been shown to be mitochondrial dependent, mediated by c-Myc, and regulated further by a balance between the anti- and pro-apoptotic proteins Bcl2 and Bax. Since we observed increasing fraction of apoptotic cells with age in spheroids, we examined the levels of these proteins. The c-Myc levels were clearly higher in spheroidal cell population as compared to monolayers, indicating that spheroids were more prone to apoptosis/necrosis under conditions of glucose deprivation. Although the levels of Bax and Bcl-2 were upregulated in the spheroids (Fig 5), the ratio of Bcl2/Bax (measured by flow cytometer), a possible regulator of the release of cytochrome c (the activator of apoptosis causing caspases), was not significantly different in both the models (data not shown).

### Radiation response

**Figure 6:**
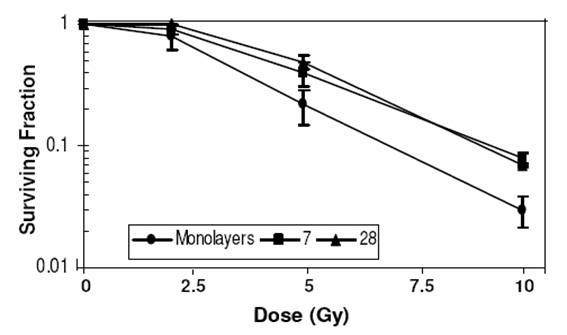
**Radiation dose response**. Radiation dose response of cell survival of BMG-1 spheroids and exponentially growing monolayer cells.

Radiation dose response was studied by analyzing the clonogenic cell survival using the macrocolony assay. Spheroids at both the ages (7 & 28 days) were found to be more resistant than the monolayer cells (Fig 6), with the values of D_q _and D_0 _as well as SF_2 _being significantly higher in the spheroids (1.8, 3.2 and 0.9 respectively on day-7) as compared to monolayers (1.2, 2.4 and 0.8) (Table 3).

### Endogenous and ionizing radiation induced ROS generation in spheroids

**Figure 7:**
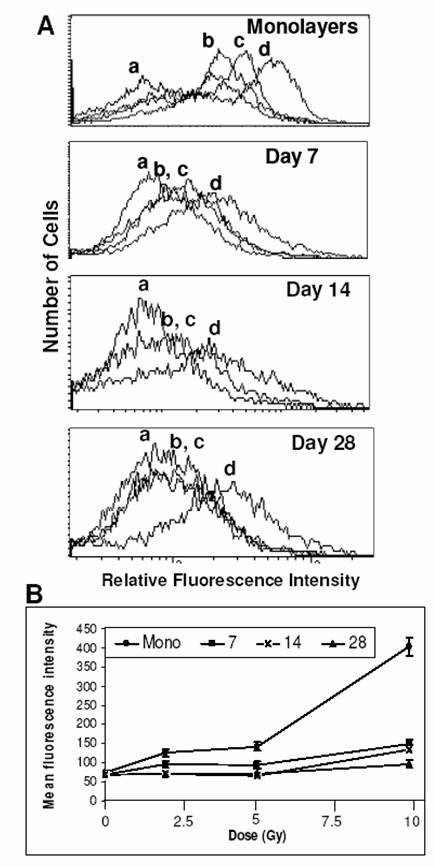
**Oxidative stress by flow cytometry**. (A) Generation of ROS measured by H_2_DCFDA technique in BMG-1 monolayers and spheroids. Untreated control (a), 2 Gy (b), 5 Gy (c) and 10 Gy (d). (B) Dose response of ROS generation in monolayers and spheroids. Average values of the mean fluorescence intensities (MFI) of H_2_DCFDA were obtained from three independent observations.

Reactive oxygen species act as mediators of radiation-induced cell damage. To determine the radiation-induced ROS generation in monolayers and spheroids, small non-necrotic as well as large spheroids containing central necrotic region were stained with the redox sensitive dye H_2_DCFDA and the fluorescence of ROS converted DCF was monitored by flow cytometry. Although endogenous levels were similar, the radiation-induced ROS generation was significantly higher in exponentially growing monolayer cells than in spheroids. The ROS levels decreased further with increase in spheroid size. Furthermore, the monolayer cells showed a bimodal distribution of values for ROS levels, while the spheroidal cell population was unimodal with wide variation in the ROS level (Fig 7A). A dose-dependent (0–10 Gy) increase in the amount of ROS was observed in the irradiated monolayers and 7-day-old spheroids (Fig 7B). However both endogenous as well as the radiation-induced ROS were reduced with increasing age of spheroids (Fig 7B). These results indicate that decrease in the levels of intracellular ROS could be a major contributing factor for radioresistance displayed by spheroids.

### Mitochondrial status

**Figure 8:**
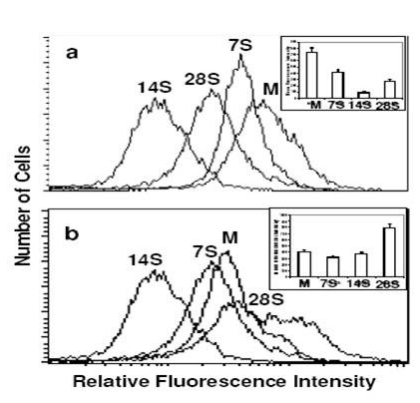
**Mitochondrial status by flow cytometry**. Flow cytometric measurements of mitochondrial mass (a) and activity (b) using NAO and Rh123 respectively in the BMG-1 spheroids and monolayers. Monolayer (M), 7-day spheroids (7 S), 14-day spheroids (14 S) and 28-day spheroids (28 S). Inset shows average values of the mean fluorescence intensities (MFI) of NAO and Rh123 obtained from three independent observations.

Mitochondria are the main site of ROS generation in the cell with 2% of oxygen consumed being leaked into cytoplasm as ROS. Since ROS generation in spheroids showed significant differences, we investigated the mitochondrial status using flow cytometric analysis following staining with NAO and Rh123. NAO accumulates in mitochondria independent of the status of mitochondrial membrane potential. Therefore, cells having more number of mitochondria will show an increase in the relative fluorescence intensity, which was collected on the log scale during flow cytometric measurements. While the MFI of monolayer cells was 740, the MFI was much lower in the 7, 14 and 28 day old spheroids, i.e., 422, 94, 274 respectively, showing a 2 to 8 fold decrease in the mitochondrial mass per cell (Fig 8a). This reduction in the number of mitochondrial mass in spheroidal cells corresponds well with reduction in ROS generation with increase in the age of spheroid.

Rh-123 is a cell-permeant cationic fluorescent dye that is readily sequestered by functionally active mitochondria having intact membrane potential. Relative fluorescence intensity of the spheroids significantly decreased significantly at 7 and 14 days as compared to the monolayers (Fig 8b). This was followed by an abrupt increase in the mitochondrial activity in the 28-day-old spheroids, although the mitochondrial mass did not increase.

## Discussion

Appropriate *in vitro *models play a vital role in understanding various molecular processes underlying tumor biology besides the development and evaluation of new therapeutic agents and strategies for improving cancer therapy. Spheroids are *in vitro *3-D model that simulates tumor micro-milieu and have been vastly used to understand different aspects of tumor biology. Therefore *in vitro *models that permit reproducible experimental studies are expected to greatly facilitate the development of newer and effective therapeutic strategies. We discuss here some of the aspects of a spheroid model developed from a human glioma cell line (BMG-1), particularly with respect to the nature and distribution of various spheroidal cell populations with increasing age and size of spheroids to understand their behaviour, particularly related to the metabolism and radiation response.

### Spheroid growth and appearance of S-phase quiescent (S_0_) cells

Spheroids are a three-dimensional cellular system that closely simulates conditions existing in the under-perfused solid tumors wherein cellular heterogeneity develops due to increasing distances from the nourishing blood capillaries [23]. In the present study, spheroids grown from an established human glioma cell line (BMG-1) showed an exponential growth pattern up to 28 days in culture, followed by rapid disintegration. While spheroids had a consistently higher proportion of G_1 _population as compared to monolayers (Table 2), their cell cycle distribution (G_0_/G_1_, S and G_2_/M phases) did not vary with growth between 7 and 28 days, except that the fraction of S-phase cells actively synthesizing DNA (S-positive cells) reduced drastically from 18% on day-7 to 5% on day-28, with the total S-phase population remaining nearly unchanged at 20% (Fig 3, Table 2). This was in contrast with the monolayer cultures wherein all the S phase cells were S positive even as they reached quiescence (Fig 3, Table 2). Cells in the spheroids are known to accumulate in a quiescent state as the growth advances [2,23,24], presumably due to micro-environmental stresses such as deprivation of growth factors [25] and/or nutrients [26] or the production of inhibitory factors [24,27] induced within the mass of spheroids. In the present study, S-phase quiescence was observed despite a consistent cell proliferation and exponential increase in spheroid volume up to 28 days (Table 1). It appears, therefore, that spheroid-specific factors like hypoxia and intercellular interactions that are different as compared to the monolayer culture mimic the conditions in large solid tumors, which result in a different type of quiescence. In contrast with the previously reported studies, we replenished the medium daily as our spheroids were unstable if not fed for long intervals of time. Regular replenishment of medium may closely resemble *in vivo *conditions in which tumor cells having sufficient vascular supply continuously receive fresh nutrients.

### Cell cycle distribution and radioresistance

It is pertinent to note here that the cell cycle distribution at the time of irradiation is an important determinant of cell survival. Cells show considerable amount of variation in the radiosensitivity as a function of their position in the cell cycle, with S phase cells being more resistant, while cells in the G_2 _and M are most sensitive. However, the relative radioresistance of spheroids (Table 3) could not be attributed to this phenomenon, as the fraction of cells in the relatively resistant S-phase was lower (~20%) in spheroids as compared to the monolayer cells (~40%). Further it is also interesting to note that the fraction of S positive cells (actively engaged in DNA synthesis, generally efficient in DNA repair) also decreased from 18% at day 7 to 5% at day 28 (Table 2) in the spheroids, while the Dq values increased from 1.8 to 2.8 under these conditions.

### Variations in cell size and cell packing density

Earlier studies have generally assumed the cell packing density to be constant throughout the growth of spheroids, and the cell population doubling time has been considered same as the volume doubling time [28]. However, our results clearly show that assuming a constant cell density may not be appropriate especially when the spheroid is cultured for long intervals of time (four weeks in this case) and/or when they are slow growing. The average cell packing density decreased from 0.36 to 0.16 with the growth of spheroids from 7^th ^day to 28^th ^day, associated with a significant increase in the average size of cells (Table 1). Therefore, the cellular load inferred from mere volume measurements during spheroid growth could be misleading.

### Incidence of cell death versus cellular radioresistance

Cell survival under conditions of hypoxic stress depends on the glycolytic status of cells [29] and the cellular resistance to hypoxia-induced apoptosis [30]. A decrease in the clonogenicity (plating efficiency) and viability was generally observed with increase in the size of spheroids (Table I), which could be associated with the induction of programmed cell death or necrosis. The Annexin-V labeling assay revealed the presence of secondary necrotic (Ann-V^+ve^/PI^+ve^) cells in the absence of apoptotic (Ann-V^+ve^/PI^-ve^) cells on days 7, 21 & 28, indicating that apoptosis may be a rather quick process in these cells, obscuring the detection of early stages.

Interestingly, although the plating efficiency was significantly reduced (25%) in the 28-day spheroids, the survival curve suggested a very high radioresistance of the surviving spheroid cells (Table 1 &3). However, these surviving cells did not belong to a specific cell-cycle phase (Table 2), implying the involvement of metabolic or other processes in radiation resistance.

### Enhanced glycolysis, hypoxia and radiation resistance

Many factors associated with hypoxia and related alterations in the metabolism have profound influence on the radiation response of cells. For example, hypoxic tumor cells generally show resistance to low LET radiation (X-rays), which has been suggested to be primarily due to the reduced radiation-induced ROS generation, responsible for the cell death through the induction of macromolecule damage, mainly in the form of DNA strand breaks [31]. However, enhanced anaerobic glycolysis, leading to accumulation of lactate may also facilitate recovery process as shown in the monolayer cells held for a few hours in high lactate medium [32,33]. In the present study, glucose utilization as well as lactate production was 2–3 fold higher in the spheroids, (Table 3) with a corresponding increase in the resistance to radiation-induced cell death (Table 3, Fig 6) as compared to the monolayer cultures. However, a distinct increase in the lactate production could not be observed in 7 day old spheroids, in-spite of Hif induction (Fig. 5), which is rather surprising and needs further investigations including the analysis of LDH. These results also suggest that, concomitant to the development of spheroid (which results in an altered cell-to-cell interaction as compared to the monolayers); there is alteration in the pattern of metabolism with further increase in glucose dependence.

Glucose usage observed in tumors often correlate with resistance to therapy and poor prognosis [34,35]. Further, we have recently observed that the radiosensitization by 2-deoxy-D-glucose (2-DG), an inhibitor of glucose transport and glycolysis, was 2–3 folds higher than in monolayers cells, which correlated well with the difference in glucose usage between the two systems [36]. This enhanced glucose usage and lactate production (anaerobic glycolysis) could be on account of compensatory increase in the constitutive level of expression of glycolytic enzymes, leading to a high rate of glycolytic capacity [37]. The activity of hexokinases is highly elevated in rapidly growing tumors as compared to normal cells [38-40]. The oxygen sensor HIF-1α also induces the expression of genes that encode glycolytic enzymes like aldolase A, enloase 1, lactate dehydrogenase A, phosphofructokinase L, phosphoglycerate kinsae 1, and pyruvate kinase M. Therefore, we investigated alterations in the levels of HIF-1α in the cells throughout the spheroid growth up to 28 days. Indeed we found a higher level of HIF-1α (Fig 5) despite daily feeding of fresh medium to the spheroids, similar to the results reported previously in the unfed spheroids [23], thereby suggesting that the enhanced glycolysis observed in spheroids could be mainly driven by hypoxia, and might be induced by the over-expression of genes encoding proteins involved in glucose metabolism.

The gene for LDH-A, whose expression is often increased in cancer cells, has been identified as a c-Myc responsive gene [41], and is also shown to be induced by hypoxia through the activity of HIF-1α [42]. Moreover, c-Myc and HIF-1α binding sites resemble the carbohydrate response element [43-45]. Cells over-expressing c-Myc have been shown to be more susceptible to the induction of apoptosis under stress induced by certain agents as well as in the sub-optimal growth conditions [46,47]. Therefore, we investigated the level of c-Myc, and indeed observed a three-fold higher level in the spheroidal cells as compared to the monolayer (Fig 5), implying that these cells might be more susceptible to apoptosis and necrosis induced by glucose deprivation.

### Mitochondrial activity and generation of reactive oxygen species

Low-LET ionizing radiation produces damage to cellular biomolecules mainly through the generation of ROS initiated by hydroxyl radicals. Apart from hypoxic cell population, enhancement of glycolysis in spheroid cells (as evident from increased glucose usage and lactate production) would result in the reduced generation of ROS following radiation exposure. Studies using confocal microscopy have shown that alterations in ATP level influences the generation of ROS in a concentration-dependent manner [48]. We did not observe any significant difference between the monolayers and spheroids of all ages with respect to the endogenous levels of ROS (Fig 7A).

The ROS generation may be reduced through partial uncoupling of oxidative phosphorylation and by decreasing the transmembrane potential and the redox state of complex 1 [49]. This is achieved by increasing permeability of the inner mitochondrial membrane to K^+ ^and H^+ ^by means of opening mitochondrial K^+ ^channels or by endogenous uncoupling proteins like carnitine palmitoyl tranferases CPT I and II [50], thereby reducing the mitochondrial membrane potential as seen in spheroids from 7 days to 21 days in culture when compared to monolayers (Fig 8b). Radiation induced ROS was 2–3 times less in the spheroids as compared to the monolayer cells at all the three absorbed doses, and this could be partly responsible for the radioresistance exhibited by the spheroidal cells (Fig 7B). However, increase in ROS most often occurs during the later stages of cell death program where cytoskeletal reorganization as well as disintegration takes place. Further, the level of intracellular ROS to a great extent determines the mode of cell death; low levels of ROS inducing PCD while high levels promotes necrosis or can lead PCD-committed cells towards necrotic-like destruction [51]. While the anti-apoptotic processes and regulators are induced to maintain the homeostasis to a certain extent (the most important family of protein being Bcl-2), alternative pathways might also be responsible for inducing apoptosis and/or necrosis.

## Conclusion

In summary, the present study provides a detailed characterization of multicellular spheroids with respect to a number of parameters relevant to studies aimed at evaluating efficacy of tumor therapeutic agents. Characterization of mitochondrial status and oxidative stress levels with the increasing age of multicellular spheroids, coupled with enhanced glycolysis and related variations in HIF-1α and c-Myc will prove useful in optimizing the use of this valuable cellular 3-D tumor model for predicting tumor response to chemotherapeutic/radiosensitizing agents acting on metabolic pathways. In this study, spheroids were regularly fed with fresh medium, which may resemble the *in vivo *conditions wherein a proportion of cells receive uninterrupted supply of nutrients and oxygen while others become hypoxic and may enter secondary necrosis. Investigations using spheroid model also highlight the inherent limitations of extrapolations made from monolayer studies for application in tumor biology and therapy. The detailed characterization presented here is aimed at obtaining a better understanding of the spheroid model for optimizing and interpreting the predictive therapy trials and drug response of various therapeutic agents and/or adjuvants, particularly metabolic modulators.

## Methods

### Monolayer culture

The cerebral glioma cell line (BMG-1; wild type p53) was established in Bangalore, India (20). Stock cultures were maintained in the exponentially growing state by passaging twice weekly in DMEM containing 10 mM HEPES and antibiotics supplemented with 5% fetal bovine serum.

### Spheroid culture

BMG-1 spheroids were grown by inoculating 1 × 10^6 ^viable cells of BMG1 in non-adherent 90-mm petridishes in 10 ml DMEM supplemented with 5% fetal calf serum, antibiotics and 10 mM HEPES. Clusters of cells could be observed after 24 hours of initiation. However, it took nearly 4 days for these clusters to form spheroids (clusters could not be dislodged by pipetting). Approximately 10,000 spheroids could be obtained in a petridish of ~60 cm^2 ^(90 mm diameter) which were redistributed in to 20 non-adherent 90-mm petridishes containing 10 ml of complete growth medium. The pH of the medium was monitored daily to prevent acidosis. The medium was changed on alternative days till spheroids were 14 day old, followed by redistribution of spheroids from each petridish further to 10 non-adherent petridishes and the medium was changed daily thereafter till 28 days. Unlike other spheroids reported in the literature these spheroids could not be grown for months as they disintegrated after 28 days.

### Spheroid growth measurements

Spheroid growth was monitored by measuring the increase in spheroid size as a function of time. Images of spheroids were obtained using the image analysis system consisting of Olympus BX 60 fluorescence microscope and Grundig FA87 monochrome CCD camera. Volume and surface area was calculated using Optimas^® ^image analysis software (Optimas, USA; version 5.0). The size of nearly 50 spheroids was evaluated in each group by measuring two orthogonal diameters (d1 and d2) using the line morphometry function. Volume was calculated using the formula V = 4/3Πr^3^, where r = 1/2√ d1d2 the geometric mean radius. Average cell number per spheroid was calculated by trypsinizing 10–20 spheroids and the total number of cells obtained was divided by the number of spheroids trypsinized.

### Analysis of cell cycle kinetics using BrdU labeling

Cells were incubated with 10 μM BrdU for 30 min at 37°C at various time intervals (7–28 days). Immediately on completion of incubation, an aliquot containing 10^6 ^cells from monolayers or spheroidal cell suspension was pelleted by centrifugation (1000 g, 10 min), resuspended in PBS, fixed in 80% ethanol and stored at 4°C until use. For analysis, fixed cells were washed with 0.9% NaCl, incubated with pepsin (0.5% in 0.055 N HCl, pH 1.8) for 10 min at 37°C, washed with saline again, and treated with 2 N HCl for 30 min at room temperature. Following another wash with normal saline and a wash with PBS-Tween (0.05%), the cells were incubated with anti-BrdU antibody (mouse anti-human IgG1; BD Pharmingen, USA) in PBS-Tween at 1:40 dilution for 30 min at 4°C. Cells were then washed twice in PBS-Tween (0.05%)-BSA (1%), and incubated with rabbit antimouse IgG1-FITC conjugate (Sigma) at 1:100 dilution in PBS-Tween-BSA for 30 min at 4°C. After another wash in PBS-Tween-BSA, cells were resuspended in PBS and stained with propidium iodide (50 μg/ml) for 30 min. Fluorescence of propidium iodide (DNA) and FITC (BrdU) was measured simultaneously in the FACS Calibur Flow Cytometer (Becton Dickinson), and biparametric scatter plots were analysed using the CellQuest software. Histograms were analyzed for cell cycle phase distribution by using Modfit software.

### Detection of PS-externalization using annexin-V labeling

Membrane asymmetry accompanied by translocation of the phospholipid phosphotidylserine (PS) from inner to the outer side of the plasma membrane is one of the manifestations of apoptosis. Externalization of PS was studied by Annexin V (a phospholipid binding protein) binding assay [22]. Briefly, live cells were washed twice in PBS and resuspended in binding buffer containing 0.01 M HEPES/NaOH, pH 7.4; 0.14 mM NaCl; 2.5 mM CaCl_2_. Cell suspension (1 × 10^5 ^cells in 100 μl) in the binding buffer was incubated with 5 μl of FITC labeled Annexin V (BD Pharmingen, USA) for 15 minutes in the dark at room temperature. Following incubation, propidium iodide was added and fluorescence of cells PI (DNA) and FITC (Annexin) was measured simultaneously in the FACS Calibur Flow Cytometer (Becton Dickinson), and biparametric scatter plots were analysed using the CellQuest software.

### Cell survival

Macrocolony assay with dissociated cell population is still considered a reference standard for assessing the survival of cells. This has been extensively employed in studies with ionizing radiation and cytotoxic anticancer drugs, to predict the dose needed to cure transplanted tumors in mice as well as multi-cellular spheroids [52,53]. Spheroids and monolayers were washed with PBS and irradiated in HBSS using Co-60 teletherapy source (Theratron-780 C, Canada) to access the cell survival. Following irradiation, spheroids and monolayer cultures were dissociated into single cells by trypsinization and counted using haemocytometer. Appropriate number of cells was plated in 60 mm Petri dishes with complete media containing 10% FCS. Plates were incubated at 37°C in a humidified incubator (5% CO_2 _and 95% air) till colonies were formed (7 days). Methanol fixed colonies was stained with 1% crystal violet and colonies containing more than 50 cells were scored.

### Estimation of glucose utilization and lactate production

BMG-1 cells and spheroids were incubated in HBSS or HBSS and 2-DG for 2 h. The amount of glucose remaining unused and the lactate produced were estimated in the buffer using enzymatic assays. Glucose was measured using the reducing sugar method [54-56]. Briefly a mixture of equal volumes of incubating medium (HBSS) and alkaline copper sulphate were incubated at 90°C for 10 minutes, followed by the addition of phosphmolybidic acid was added at room temperature (~27°C). O.D of the resulting chromogen was measured at 540 nm. Lactate was estimated using lactate oxidase method based kit (Randox; cat No.-LC2389). The number of viable cells in spheroids was counted and glucose consumed or lactate produced was normalized with respect to number of viable cells.

### Determination of intracellular redox levels

Intracellular redox levels were measured using the fluorescent dye H_2_DCFDA which is a non-polar compound (Merck England) and yields a fluorescent DCF in the presence of ROS in the cells. Green fluorescence due to intracellularly trapped DCF was collected on the FL1 channel on the log scale. Spheroids and monolayer cells were just washed twice in PBS and held in PBS with Ca^2+^, Mg^2+^, H_2_DCFDA (10 μg/ml) and 5 mM glucose, before irradiation and subsequently incubated for half an hour at 37°C. Cells were trypsinized after a rinse with PBS, washed and resuspended in PBS with Ca^2+^, Mg^2+ ^and glucose. Samples were stored on ice, and measurements were made within half an hour after trypsinization.

### Flow cytometric analysis of mitochondrial mass and activity

Spheroids and monolayers were parallely stained with one of the mitochondrial fluorescent dyes NAO and Rh123, essentially according to the procedure described earlier [57]. Briefly, the unfixed spheroids and monolayers were washed and incubated in PBS (with Ca^2+^, Mg^2+^), glucose and NAO (10 μM 10 min at 25°C) or Rh123 (5 μg/ml 30 min. at 37°C), followed by trypsinization washing and resuspension in PBS (with Ca^2+^, Mg^2+ ^and glucose) and incubated on ice until analysis by the flow cytometer.

### Preparation of cell extract and western blotting

Scraped monolayer cells and spheroids were collected at 800 g for 5 minutes, washed twice with 5 ml PBS and sonicated (VC-X 500, Sonics and Materials Inc. USA) on ice in PBS for 1 minute mixed with pre chilled acetone (3 parts) and incubated overnight at -20°C. Following incubation, acetone was removed by centrifugation and pellet was dissolved in autoclaved Milli Q water and protein content was determined by Lowry's method [58]. 2× lysis buffer [62.5 mM Tris Hcl ph 6.8, 10% (v/v) Glycerol 2% (w/v) SDS, 1 mM PMSF, 1 μg/ml pepstatin A, 1 μg/ml leupeptin and 5 μg/ml aprotinin] was added to the pellet and boiled at 100°C for 5 minutes. An equal amount of protein (10–20 μg) was loaded on 10% SDS polyacrylamide gels. Proteins were separated at a constant voltage of 100 V for 1.5 hours and then blotted to PVDF membrane (Amersham) in transfer buffer [25 mM Tris, 192 mM glycine, 15% methanol (v/v)] overnight at 14 V. The blots were blocked with 1% BSA in TTBS [15 mM Tris-HCl pH7.5, NaCl .9% and 0.1% Tween-20] for 1 hour at room temperature (25–28°C). Membranes were incubated with a mouse monoclonal antibody (1:1000) anti Bax (BD Pharmingen, USA), anti Bcl2 (BD Pharmingen, USA) anti c-Myc (BD Pharmingen, USA), anti HIF-1α (Santa Cruz Biotechnology USA) and anti Beta actin (Promega) for 1 hour in TTBS and 1% BSA. Membranes were washed with TTBS four times for 15 minutes each and incubated with anti mouse peroxidase conjugated secondary antibody (1:1000 dilutions; Banglore Genei India) for 1 hour. Blots were washed and developed using ECL chemiluminescence detection reagent (Pierce). Membranes were stripped in stripping buffer (25 mM Glycine, 1% SDS, pH 2 adjusted with HCl) for 1 hour washed twice in TTBS for 10 minutes each and re-probed.

## Authors' contributions

DK contributed 50% in all aspects of this work. SC helped in the microscopy and helped to draft the manuscript. MBA helped in the enzymatic assays and statistical analysis of the data. BSD participated in design and coordination of the study and helped to draft the manuscript. All authors read and approved the final manuscript.
